# A Study of Factors Predicting Difficulties in Transesophageal Echocardiography (TEE) Probe Insertion in Adult Patients Undergoing Cardiac Surgery

**DOI:** 10.7759/cureus.64256

**Published:** 2024-07-10

**Authors:** Shahbaz Hasnain, Arpith Shenava, Ipshita Garg

**Affiliations:** 1 Anaesthesiology, Dr. D. Y. Patil Medical College, Hospital & Research Centre, Pune, IND

**Keywords:** patient safety, jaw thrust manoeuvre, bmi, cormack-lehane grading, mallampati score, airway assessment, predictive factors, cardiac surgery, tee probe insertion, transesophageal echocardiography

## Abstract

Background and objective

While transesophageal echocardiography (TEE) is crucial in cardiac surgery, the probe insertion can be challenging. This observational study aimed to identify predictive factors associated with difficult TEE probe insertion in adult cardiac surgery patients.

Methods

A total of 119 adult patients undergoing cardiac surgery were included in the study. Demographic variables (age, gender, and BMI) and airway factors (modified Mallampati classification, modified Cormack-Lehane grading, and thyromental distance) were analyzed. The difficulty of TEE probe insertion was categorized into three grades, and various maneuvers were assessed for difficult insertions.

Results

Of note, 30.3% of insertions were difficult. Male gender (OR: 1.8), BMI ≥30 kg/m^2^ (OR: 2.5), Mallampati class III-IV (OR: 3.2), Cormack-Lehane grade IIb-IV (OR: 2.7), and thyromental distance <6.5 cm (OR: 1.9) were significantly associated with difficult insertion. Jaw thrust was the most effective maneuver (58.3%) for difficult cases.

Conclusions

Based on our findings, several demographic and airway factors predict difficulties in TEE probe insertion. Understanding these factors can help clinicians anticipate challenges and prepare appropriate strategies, potentially reducing complications associated with probe insertion.

## Introduction

Transesophageal echocardiography (TEE) is a widely adopted imaging modality in cardiac surgery, offering real-time, high-resolution images of the heart and its surrounding structures. It plays a crucial role in guiding surgical decision-making, monitoring procedural progress, and assessing postoperative outcomes [1]. However, despite its significant clinical utility, the insertion of the TEE probe can be challenging, posing potential risks of complications such as oropharyngeal injuries, esophageal perforation, dysphagia, and gastrointestinal bleeding [2]. The incidence of these complications is further exacerbated when using larger real-time 3D TEE probes, which are increasingly preferred for their enhanced imaging capabilities [3]. Furthermore, certain demographic and anatomical factors may contribute to the difficulty of probe insertion, including age, gender, BMI, and airway characteristics [4].

Previous studies have highlighted the impact of high Mallampati scores, Cormack-Lehane grades, and thyromental distance on the ease of TEE probe insertion [5]. These parameters are commonly used in predicting difficult endotracheal intubation, suggesting a potential correlation with challenging TEE probe placement [6]. To mitigate the risks associated with repeated blind attempts at probe insertion, various techniques have been proposed, such as the jaw-thrust maneuver, reverse Sellick's maneuver, direct laryngoscopy, and video laryngoscopy [7]. While these maneuvers may improve the success rate and reduce complications, their routine implementation may not be feasible in all clinical settings, particularly outside the operating room environment [8]. Given the increasing reliance on TEE in cardiac surgery and the potential risks associated with its insertion, it is crucial to identify predictive factors that can aid in anticipating and managing difficult probe placements. By understanding the demographic and anatomical characteristics that contribute to insertion challenges, clinicians can better prepare and employ appropriate strategies to ensure safe and successful TEE probe insertion [9].

This observational study aims to investigate the predictive factors associated with difficulty in TEE probe insertion in adult patients undergoing cardiac surgery. By analyzing demographic variables (gender and BMI) and airway factors (Mallampati scores, Cormack-Lehane grading, and thyromental distance), the study seeks to quantify the contribution of each factor to the overall difficulty encountered during probe insertion. Additionally, the study will assess the specific maneuvers employed in cases of difficult insertion, providing valuable insights into the efficacy and applicability of various techniques.

## Materials and methods

This observational study was conducted in a third-tier institute over one year. Ethical approval was obtained from the institutional ethics committee before the commencement of the study. All subjects underwent a thorough pre-anesthetic evaluation, and relevant laboratory investigations were performed. Informed written consent was obtained from all patients. Our reference study was “Factors predicting difficulty in the insertion of real-time-three-dimensional transesophageal echocardiography probe in adult patients undergoing cardiac surgery” by Kiran et al. [3]; based on the proportion of patients having difficulty in probe placement as (0.27 as cited in the article), with the acceptable difference of 0.08 with 95% confidence level, the minimum sample was calculated to be 119 using software WINPEPI app version 11.3.

The study included 119 adult patients aged 18-75 years who were scheduled for cardiac surgeries. Patients with ASA grade II and III who were hemodynamically stable with all routine investigations within normal limits were included in our analysis. Patients with restricted mouth opening and/or neck extension (<30 degrees) or those with absolute and relative contraindications to the TEE probe placement were excluded. Absolute contraindications included patients with a history of esophagectomy, and patients having esophageal stricture, tumor, esophageal diverticulum, or esophageal perforation or laceration. Patients having active upper GI bleed or those planned for operations involving the pharynx, esophagus, or stomach also were excluded. Relative contraindications included patients with a history of radiation to the neck and mediastinum, or patients having Barrett’s esophagus, active esophagitis, esophageal varices, symptomatic hiatal hernia, and active peptic ulcer disease. Also, those with known or suspected cervical spine injury or any coagulopathy or thrombocytopenia were excluded.

Patients were brought into the operating room, and standard monitoring equipment was attached. After routine anesthesia induction and muscle relaxation, the trachea was intubated with appropriately sized endotracheal tubes. A well-lubricated TEE probe was then inserted by a senior anesthesiologist. The distal 10 cm of the probe was generously lubricated with water-soluble jelly on all sides. For patients not requiring laryngoscopy, a bite block was placed first, followed by the application of 2% lignocaine jelly inside the block before probe insertion. In cases requiring laryngoscopy, the bite block was slid over the probe after its insertion. The patient's head was positioned in the sniffing position, elevated by 10 cm from the operating table using folded sheets, bringing the ear canal to the level of the anterior shoulder.

If initial attempts to insert the TEE probe into the esophagus failed, upper airway manipulations were attempted. These manipulations included jaw thrust, reverse Sellick's maneuver, and direct laryngoscopy, in that order. Video laryngoscopy-guided insertion was used as a final resort if all other maneuvers failed. The difficulty of TEE probe placement was categorized into three grades: grade 1 (requiring jaw thrust), grade 2 (requiring reverse Sellick's maneuver), and grade 3 (requiring direct laryngoscopy). Predictive factors related to difficult endotracheal intubation were evaluated for TEE probe placement. These factors included age, gender, BMI, modified Mallampati classification, modified Cormack-Lehane grading, and thyromental distance. The primary outcome was the calculation of the percentage of difficulty contributed by demographic and airway factors in inserting the TEE probe. The secondary outcome was the assessment of the maneuvers used in cases of difficult TEE probe insertion.

Data were collected using a preformed data collection form and entered into Excel for analysis. Statistical analysis was performed using appropriate software such as Epiinfo, SPSS Statistics, or Medcalc. Qualitative data were summarized using proportions/percentages, and quantitative data was presented as mean and standard deviation (SD). Appropriate tests of significance, such as chi-square, were performed. Qualitative data were analyzed using thematic analysis. A p-value less than 0.05 was considered statistically significant.

## Results

Table 1 provides an overview of the study population. The average age of participants was 62.5 years, which is typical for cardiac surgery patients. There was a higher proportion of males (61.3%) compared to females (38.7%), reflecting the general trend in cardiac surgery demographics. The BMI distribution showed that a significant portion of patients were overweight (40.3%) or obese (16.0%), potentially impacting TEE probe insertion difficulty.

**Table 1:** Demographic characteristics of the study participants BMI: body mass index; SD: standard deviation

Variable		Values
Age, years, mean ± SD		62.5 ± 9.8
Gender, n (%)	Males	73 (61.3%)
	Females	46 (38.7%)
BMI, kg/m², n (%)	Underweight (<18.5)	5 (4.2%)
	Normal (18.5-24.9)	47 (39.5%)
	Overweight (25-29.9)	48 (40.3%)
	Obese (≥30)	19 (16%)

Table 2 outlines the airway characteristics that might affect the TEE probe insertion. A considerable number of patients had higher Mallampati scores (class III and IV totaling 40.4%), which could predict difficult probe insertion. The Cormack-Lehane grading showed that 26% of patients had grade IIb or higher, potentially indicating challenging airways. Most patients (77.3%) had a thyromental distance ≥6.5 cm, which is generally considered favorable for airway management.

**Table 2:** Airway characteristics of the study participants

Characteristic		N (%)
Modified Mallampati classification	Class I	28 (23.5%)
	Class II	43 (36.1%)
	Class III	36 (30.3%)
	Class IV	12 (10.1%)
Modified Cormack-Lehane grading	Grade I	51 (42.9%)
	Grade IIa	37 (31.1%)
	Grade IIb	19 (16 %)
	Grade III	10 (8.4%)
	Grade IV	2 (1.6%)
Thyromental distance	<6.5 cm	27 (22.7%)
	≥6.5 cm	92 (77.3%)

Table 3 shows the distribution of TEE probe insertion difficulty. While the majority of insertions (69.7%) were performed without difficulty, 30.3% required some form of intervention. Of these, most were resolved with jaw thrust (17.6%), fewer needed reverse Sellick's maneuver (9.2%), and only a small percentage required direct laryngoscopy (3.4%).

**Table 3:** Difficulty in TEE probe insertion TEE: transesophageal echocardiography

Difficulty level	N (%)
No difficulty	83 (69.7%)
Grade 1 (jaw thrust)	21 (17.6%)
Grade 2 (reverse Sellick's)	11 (9.2%)
Grade 3 (direct laryngoscopy)	4 (3.4%)

Table 4 presents the odds ratios for various factors associated with difficult TEE probe insertion. All factors showed statistically significant associations (p<0.05). Notably, Mallampati class III-IV had the strongest association (OR: 3.2), followed by Cormack-Lehane grade IIb-IV (OR: 2.7) and BMI ≥30 (OR: 2.5). Male gender and thyromental distance <6.5 cm also showed significant associations but to a lesser extent.

**Table 4:** Association of various factors with difficult TEE probe insertion BMI: body mass index; TEE: transesophageal echocardiography

Factor	Odds ratio	95% confidence interval	P-value
Male gender	1.8	1.1-2.9	0.02
BMI ≥30 kg/m^2^	2.5	1.4-4.5	0.002
Mallampati class III-IV	3.2	1.9-5.4	<0.001
Cormack-Lehane grade IIb-IV	2.7	1.6-4.6	<0.001
Thyromental distance <6.5 cm	1.9	1.2-3.1	0.01

Table 5 details the maneuvers used in cases of difficult insertion. In difficult insertions, jaw thrust was the most commonly used and effective maneuver (58.3%), followed by reverse Sellick's maneuver (30.6%). Direct laryngoscopy was needed in 11.1% of difficult cases. Interestingly, video laryngoscopy was not required in any of our cases, suggesting that the other techniques were sufficient to overcome insertion difficulties.

**Table 5:** Maneuvers used for difficult TEE probe insertion TEE: transesophageal echocardiography

Maneuver	N (% of difficult insertions)
Jaw thrust	21 (58.3%)
Reverse Sellick's	11 (30.6%)
Direct laryngoscopy	4 (11.1%)
Video laryngoscopy	0 (0%)

## Discussion

Our study aimed to identify factors associated with difficult TEE probe insertion in adult cardiac surgery patients. Both demographic and airway factors contribute significantly to insertion difficulty. The incidence of difficult TEE probe insertion in our study was 30.3%, which aligns with the findings of Kiran et al. (27.5%) [3]. This similarity suggests a consistent rate of difficulty across different populations undergoing cardiac surgery. We found that male gender was associated with increased difficulty in TEE probe insertion (OR: 1.8, 95% CI: 1.1-2.9, p=0.02). This aligns with the findings of Godoy et al., who reported that the male gender is associated with increased tongue adiposity, potentially complicating airway management [10]. However, Chang et al. did not find a significant gender difference in their study [7], highlighting the need for further investigation into this factor. Our results showed a strong association between BMI ≥30 and difficult TEE probe insertion (OR: 2.5, 95% CI: 1.4-4.5, p=0.002). This is consistent with the findings of Kiran et al., who reported that obesity was a significant predictor of difficulty [3]. The increased difficulty in obese patients may be attributed to excess pharyngeal soft tissue and reduced upper airway space.

In our study, Mallampati class III-IV showed the strongest association with difficult TEE probe insertion (OR: 3.2, 95% CI: 1.9-5.4, p<0.001). This corroborates the findings of Khongkaew et al., who reported that higher Mallampati scores were positively associated with longer TEE probe insertion times [8]. Similarly, we found that Cormack-Lehane grade IIb-IV was significantly associated with difficulty (OR: 2.7, 95% CI: 1.6-4.6, p<0.001), which is in line with the findings of Na et al. [6].

Regarding the maneuvers used to overcome insertion difficulties, our study found that jaw thrust was the most frequently used and effective technique (58.3% of difficult cases). This aligns with the findings of Chang et al., who reported that the jaw-thrust maneuver facilitated TEE probe insertion and reduced oropharyngeal injury [7] However, unlike the study by Ozturk et al. [9], we did not need to use video laryngoscopy in any case, suggesting that simpler techniques were sufficient in our population.

Our findings also align with recent literature on the subject. Rao et al. conducted a prospective observational study and found that modified Mallampati grade III/IV, neck circumference >40 cm, and limited neck extension were independent predictors of difficult TEE probe insertion [11] This further emphasizes the importance of comprehensive preoperative airway assessment in anticipating difficulties. Interestingly, a study by Ning et al. explored the use of ultrasonography for predicting difficult TEE probe insertion. They found that the retropharyngeal space diameter measured by ultrasound was a significant predictor of difficulty [12]. While our study did not utilize ultrasonography, this approach could be considered in future research to enhance prediction accuracy.

The potential complications associated with difficult TEE probe insertion should not be underestimated. Iriarte et al. reported a case of esophageal perforation following TEE probe insertion in a patient with undiagnosed Zenker's diverticulum [13]. This highlights the importance of a thorough preoperative evaluation and careful insertion techniques, especially in patients with identified risk factors. A systematic review by Mathur et.al. examined various methods to facilitate TEE probe insertion. They concluded that while multiple techniques exist, including the use of video laryngoscopy and ultrasound guidance, simple maneuvers like jaw thrust remain effective first-line approaches [14]. Our findings support this conclusion, as jaw thrust was successful in the majority of our difficult cases. Lastly, Sawasdiwipachai et al. investigated the learning curve for TEE probe insertion among anesthesia residents. They found that proficiency typically required about 30 supervised insertions [15]. This underscores the importance of proper training and experience in performing this procedure, particularly in patients with identified risk factors for difficult insertion.

Our study has some limitations. As an observational study, it cannot establish causality. Additionally, the single-center design may limit the generalizability of our findings. Future multi-center studies with larger sample sizes could provide more robust evidence.

## Conclusions

Our study identifies several predictive factors for difficult TEE probe insertion in cardiac surgery patients, including male gender, obesity, high Mallampati scores, and unfavorable Cormack-Lehane grades. The jaw-thrust maneuver proved to be an effective technique in managing the most difficult insertions. These findings can help clinicians anticipate challenges and prepare appropriate strategies, potentially reducing complications associated with probe insertion. Furthermore, our results emphasize the importance of comprehensive preoperative airway assessment and the value of simple, effective maneuvers in overcoming insertion difficulties. Future research should focus on validating these findings in larger, multi-center studies and exploring additional predictive tools, such as ultrasonography, to further enhance patient safety during TEE probe insertion.
